# Algal Cell Response to Pulsed Waved Stimulation and Its Application to Increase Algal Lipid Production

**DOI:** 10.1038/srep42003

**Published:** 2017-02-10

**Authors:** Oleksandra Savchenko, Jida Xing, Xiaoyan Yang, Quanrong Gu, Mohamed Shaheen, Min Huang, Xiaojian Yu, Robert Burrell, Prabir Patra, Jie Chen

**Affiliations:** 1 Department of Biomedical Engineering, University of Alberta, Edmonton, Canada; 2 Department of Electrical and Computer Engineering, University of Alberta, Edmonton, Canada; 3 Department of Biomedical Engineering, University of Bridgeport, Connecticut, USA; 4 Department of Mechanical Engineering, University of Bridgeport, Connecticut, USA

## Abstract

Generating renewable energy while sequestering CO_2_ using algae has recently attracted significant research attention, mostly directing towards biological methods such as systems biology, genetic engineering and bio-refining for optimizing algae strains. Other approaches focus on chemical screening to adjust culture conditions or culture media. We report for the first time the physiological changes of algal cells in response to a novel form of mechanical stimulation, or a pulsed wave at the frequency of 1.5 MHz and the duty cycle of 20%. We studied how the pulsed wave can further increase algal lipid production on top of existing biological and chemical methods. Two commonly used algal strains, fresh-water *Chlorella vulgaris* and seawater *Tetraselmis chuii*, were selected. We have performed the tests in shake flasks and 1 L spinner-flask bioreactors. Conventional Gravimetric measurements show that up to 20% increase for algal lipid could be achieved after 8 days of stimulation. The total electricity cost needed for the stimulations in a one-liter bioreactor is only one-tenth of a US penny. Gas liquid chromatography shows that the fatty acid composition remains unchanged after pulsed-wave stimulation. Scanning electron microscope results also suggest that pulsed wave stimulation induces shear stress and thus increases algal lipid production.

Oleaginous plants, often food crops, store large amounts of lipids in seeds and provide energy for plant growth during germination^1^. However, to produce biofuel on a commercial scale, a controversial “food vs. fuel” issue arises. The United States Department of Energy launched the Aquatic Species Program (ASP) in 1978 to develop renewable fuel for transportation. The program focused on producing biodiesel fuel from algae and reached the following conclusions regarding the impact of algae on resources:

1. Microalgae produce lipids with high efficiency – achieving yields up to 80% of the dry biomass using a photosynthetic mechanism for CO_2_ sequestration^2^,^3^.
2. Microalgae absorb greenhouse gases responsible for climate change^4^. They produce lipid oils at efficiencies 30 to 100 times higher than edible agricultural products, such as corn and soybeans – classified as first-generation biofuel sources^5^.
3. Microalgae preserve arable soils^6^ and other natural resources^7^, and need only marginal lands for cultivation.

In addition to CO_2_ sequestration and algal oil generation, microalgae can also be used in wastewater treatment^8^. Wastewater contains organic carbon, nitrogen and phosphate, which support the growth of many species of microalgae in heterotrophic or mixotrophic growth conditions. Microalgae can use wastewater as its growth medium and the production of algal oils is biodegradable and non-toxic^9^. Algal consumption of organic carbon, nitrogen and phosphate salts decreases the biological oxygen demand (BOD) in wastewater and thus makes biodiesel fuel production from algae economically feasible^6^,^10^. Pacific Northwest National Laboratory recently reported that crude algae oil was converted into aviation fuel, gasoline or diesel fuel^11^. Various studies conclude that microalgae hold great potential as the next generation sustainable source of renewable energy for the next 10 to 15 years^12^,^13^,^14^ with appropriate fuel grade algae growth. Recent drop in oil prices poses a short-term challenge for biofuel production, by rendering the production price of biodiesel more expensive than oil price on the market. While this trend may be temporary, biofuel production will still need federal support to make sure the industry survives at times when oil prices drop significantly. According to the implementation and finalization of the “Clean Power Plan” drafted by United States Environmental Protection Agency in August 2015, support for renewable energy becomes a global strategy in order to cut down green gas emissions.

Microalgae can grow in phototrophic, heterotrophic, or mixotrophic conditions^15^. In phototrophic growth conditions, carbon arising from greenhouse gases (CO_2_) is present in cells. Microalgae efficiently convert CO_2_ into algal oil, a precursor to biodiesel fuel. One hundred tons of algae can capture 180 tons of CO_2_ by photosynthesis^16^. Phototrophic growth is an elegant conversion pathway, but it is not economically feasible for commercialization^17^. Industries generally favor heterotrophic growth conditions due to the faster production of cell biomass and oil content. For instance, the heterotrophic growth of *Chlorella protothecoides* can accumulate lipid concentrations as high as 55% of the cell dry mass after 144 hours of cultivation with a feedstock of corn powder hydrolysate^18^. Mixotrophic growth conditions have been reported to be even better than heterotrophic growth alone in terms of biomass productivity as well as algal oil content^15^.

Microalgae are not naturally adapted to biofuel production. Its function involves complex regulatory networks and mutual interaction of physiological processes and organelles. To identify the optimal strategy to increase algal oil production, a systems biology approach, in particular *in silico* model, was used to identify genetic and physiological modification^19^. Molecular toolkits were also used to prepare various algal strains most suitable for lipid production^20^. In addition, methods to improve light absorption and photo-synthetic efficiency and to improve carbon fixation were reported in^21^,^22^ and^23^, respectively. Because microalgae involve complex lipid metabolism, different metabolic engineering approaches were proposed to improve lipid production^24^,^25^.

Microalgae do not directly produce biofuel, but they produce lipids, such as triacyl-, diacyl- and monoacyl-glycerols, which can be transformed in methanol esters to be used as biofuel. Despite improvements in optimizing microalgal growth, the price of biofuel is approximately 1.5 to 2 times higher than that of conventional fossil fuel^17^. The search for growth conditions (nutrition, temperature, oxygen level) that maximize algal biomass productivity and oil storage continues^26^. Stress responses of lipid metabolism in the oleaginous microalgae have drawn increasing research attention. Increasing lipid production in the context of stress exposure, such as N-availability, temperature, high irradiance and salinity was also reported^27^. The growth of *Nannochloropsis* in nitrogen-replete media under a high light condition triggered a rapid acclimation of the microalgae to the high light stress in a salinity-dependent manner associated with a moderate decrease in eicosapentaenoic acid proportion of total fatty acid. The influence of microwave radiation on physiological changes (higher growth rates and biomass) was reported in^28^. The microwave power of 750 W was found to be the optimum microwave power as the maximum bio-oil yield of 28.6% was obtained^29^.

In this article, we present a mechanical wave instead of microwave, specifically pulsed wave, to improve algal lipid production efficiency. Two commonly used fresh water and seawater microalgae*, Chlorella vulgaris* and *Tetraselmis chuii*, were chosen.

## Materials and Methods

### Chemicals

Mowiol^®^ 10–98, polyvinyl alcohol (PVA) with a molecular weight (M_w_) of 61000, 99% linolenic acid, and Nile Red dye were purchased from Sigma-Aldrich (St. Louis, MO). BODIPY 505/515 fluorescent molecular probe was purchased from Invitrogen (Carlsbad, CA).

### Algae preparation

*C. vulgaris*, fresh water green algae was grown in a modified 3 N bold medium. The standard recipe of 3 N bold medium was used for algal growth medium^30^. However, the amount of nitrogen (NaNO_3_) was decreased 3-fold (from 75 g/L of NaNO_3_ in stock solution to 25 g/L). *T. chuii*, a seawater green algae was grown in F/2 medium^31^, prepared using concentrated F/2 medium from the kit (Bigelow Laboratory for Ocean Sciences) and artificial seawater (Tap water containing sea salt mixture from “Instant Ocean” at 35 g/L. “Instant Ocean” refers to a commonly used brand of marine salt). Filter sterilization was used to avoid precipitation. The total growth cycle was 8 days.

### Culture conditions

The algal strains were grown mixotrophically in a shake flask at 27 °C with a shaking speed of 150 rpm. 1 L of filter-sterilized medium was inoculated with exponentially growing seed culture at a concentration of 10% of the total medium. Spectrophotometry at the optical wavelength of 550 nm (OD_550_) was used, revealing that the OD measurements of the initial solution were between 0.09 and 0.1. The initial solution was divided between flasks for ultrasound treatments under different conditions. The culture in the shake-flasks was exposed to light flux - 60 μmol photons/m^2^/sec for 16-hours (light on), and 8-hours of lights off (darkness). Algae were grown for a total of 8 days. 15 mL of glucose (40% solution) was added to the culture (100 mL) on the 5^th^ day of the growth cycle so that the final concentration was 6% glucose in the flask. Daily measurements of OD_550_ were performed to check the cell growth. Lipid accumulation was also tested daily using the developed technique with fluorescent lipid-staining probe and flow cytometry. At the end of cultivation, the culture was harvested with centrifugation at 5000 rpm and freeze-dried for further lipid extraction.

### Pulsed-wave generating device

**Figure 1:**
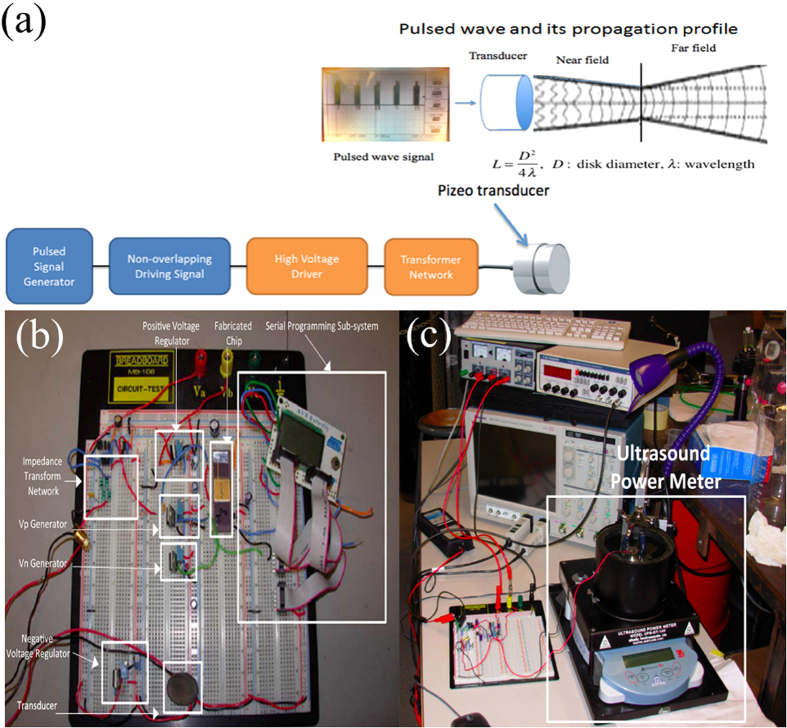
**(a)** LIPUS design consists of various modules: such as pulsed signal generation, signal amplification and impedance matching for driving piezoelectric transducers, (b) the detailed LIPUS design was mapped on a breadboard with individual components marked, (c) the ultrasound power meter was used to calibrate the output intensities of pulsed wave.

Current research on increasing algal oil production primarily focuses on algae strain selection, genetically modifying algae, as well as the optimization of medium and growth conditions^32^,^33^. Unlike the previously mentioned biochemical approaches, we have developed a physical stimulation method, or a pulsed wave known as low-intensity pulsed ultrasound (LIPUS) with frequency of 1.5 MHz, duty cycle of 20%, and intensity within 20 mW/cm^2^–200 mW/cm^2^ to increase lipid accumulation in algae. The system-level design, the board-level design and the set-up for ultrasound calibration are shown in Fig. 1(a–c), respectively.

### Experimental Set-up

**Figure 2:**
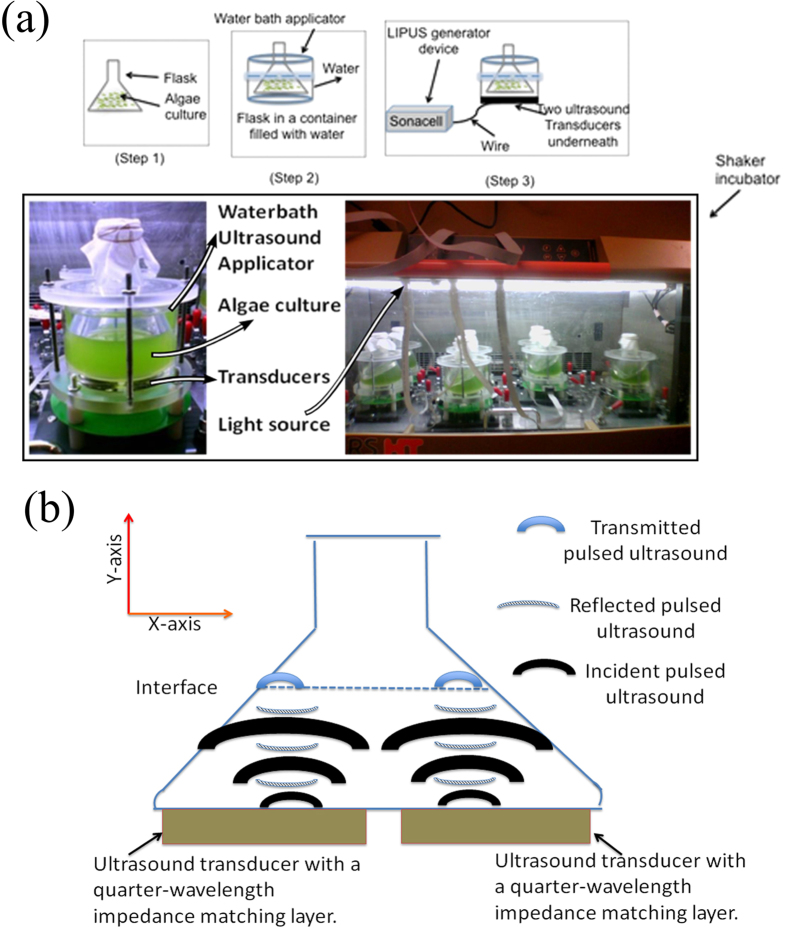
(**a**) Experimental setup for algae cultivation in a shaker incubator with proper lighting. **(b)** Overall schematic image of shake flask in water bath. **(c)** Experimental setup for algae cultivation in spin-flask bioreactors.

The experimental setup of our proposed pulsed wave stimulation is as follows. A pulsed wave device, more specifically a low-intensity pulsed ultrasound (LIPUS) device with 1.5 MHz and 20% duty cycle, was designed to generate the required pulsed wave for the experiments. The profile of the pulsed wave is shown in Fig. 1a. The waves were applied to the culture by placing the culture flask in a customized water-bath applicator, which was designed specifically for individual flasks (refer to Fig. 2(a)). In the water bath applicator, two ultrasound transducers were mounted underneath because one transducer cannot cover the bottom area of the flask. Note that ultrasound can propagate efficiently through aqueous solutions, but not through air. Therefore, the flask was subsequently placed in the water bath applicator (step 2 in Fig. 2(a)) where water serves as a propagating medium. In this case, ultrasound can efficiently penetrate without attenuation in the Y-axis (refer to Fig. 2(b)). In addition, the flask was shaken at 150 rpm during cell culturing. Statistically, cells receive equal sonication. Overall, ultrasound waves penetrate uniformly through the bottom of the flask to reach the cell culture.

These applicators together with flasks were placed into the shaker incubator (step 4 in Fig. 2(a)). The incubator was equipped with a controlled light source (16-hour light on and 8-hour light off per day) and special holders on the floor of the shaker incubator to secure the applicators in place during shaking. The shaker was set at 150 rpm, with temperature controlled at 27 °C during the whole cell growth cycle. Different samples received different intensities of ultrasound stimulation (e.g. control without stimulation, 60 mW/cm^2^, 80 mW/cm^2^ and 100 mW/cm^2^) during the cell growth cycle. The intensity used in the experiment refers to the intensity of spatial peak temporal average, or I_SPTA_ = 40, 60 and 80 mW/cm^2^ (or ultrasound pressure amplitude of 268.76, 403.15, and 537.53 mPa) applied to cell stimulation. They were individually calibrated using an ultrasound power meter (Ohmic Instruments Company, Maryland, USA). Figure 2(c) shows the experimental setup for algae cultivation in spin-flask bioreactors.

### Algal cell lipid staining

Nile Red dye (9-diethylamino-5Hbenzo [α] phenoxazine-5-one) was prepared as a stock solution at a concentration of 0.25 mg/mL in acetone. Stock solutions of the dye were kept in the dark at −20 °C. Staining of the *C. vulgaris* cells was performed using a technique^34^ where glycerol solution served as a carrier for the dye. *Chlorella vulgaris* cell culture was diluted 10 times using deionized (DI) water. 3 mL of algae suspension was mixed with 0.8 mL of the glycerol solution (0.5 g/mL) so that the final glycerol concentration was 0.1 g/mL. 20 μL of Nile Red in acetone was added to the samples, which were subsequently vortexed for 1 minute and left in the dark for 10 minutes. To improve staining efficiency, samples of 1 mL were then sonicated for 30 minutes and heated to 60 °C in a water bath for 30 minutes. *C. vulgaris* cells have a thick cell wall rendering it very difficult to stain the cells. Because only 1 mL of sample was used, higher staining temperature could be applied and the sample was then disposed. After numerous trials, we discovered that 60 °C plus sonication could achieve a staining rate of 97 to 100% while keeping cells intact. Unbound dye was removed by centrifugal washing for 5 minutes at 1500 rpm. This procedure was performed three times. For BODIPY staining 3 mL of cell suspension was mixed with 20μL of 1 mM BODIPY in dimethyl sulfoxide (DMSO) solution, vortexed for 1 minute, and then sonicated for 35 minutes at 60 °C.

*T. chuii* samples were tested without dilution because their concentration was much lower than that of *C. vulgaris* samples (around 1×10^6^ cells/mL). Centrifugal washing was not applied because the change in osmotic pressure would cause cell rapture. Nile red staining was performed in the same way as for *C. vulgaris*, though without sonication because *T. chuii* has thin cell wall allowing dye to easily penetrate into the cell body. The sample was heated to 40 °C for 1 hour. For the BODIPY staining, 3 mL of cell suspension was mixed with 20 μL of 1 mM BODIPY in DMSO solution, vortexed for 1 minute, and then kept in the dark for 10 minutes.

### Emulsion preparation and particle staining

Linolenic acid was used as the oil phase in an oil/water emulsion. 5 mL of 6% w/v solution PVA was used as the water phase. PVA also served as a surfactant. A fixed amount (20 μL) of linolenic acid was used in the flow cytometry experiment. The mixtures were emulsified using a high-speed homogenizer (Fisher Scientific PowerGen, Model 125) for 40 minutes at 5 different speeds in the range 20000–30000 rpm for different sizes (within 2.8–3.7 μm range) of the oil droplets. Particle size was estimated by the Dynamic Light Scattering (DLS, Malvern, Zetasizer Nano S). Stability of the obtained emulsions was tested using the spectrophotometer. Particles in the emulsion were stained using both NR and BODIPY in the same way as algae cells: 20 μL of dye solution (NR in acetone or BODIPY in DMSO) was added to 3 mL of emulsion, followed by 1 minute of vortexing, and kept for 10 min in the dark.

### Measurements of fluorescence

Fluorescence measurements for both lipid particles in the emulsion and lipids inside the cells were performed using a flow cytometry apparatus with an argon ion laser (BD FACS Calibur, Flow cytometer, Becton Dickinson). The FL2 (orange-red) channel was used for Nile Red fluorescence intensity measurements with excitation at 488 nm. Green fluorescence of the BODIPY was detected using the FL1 (green) channel with an excitation wavelength of 503 nm.

### Gravimetric lipid extraction

Algae cells from the culture were collected into tubes, centrifuged and freeze-dried using a vacuum system for 24 hours. Dry biomass of each sample was mixed with a solvent composed of Chloroform:Methanol = 2:1^35^. 5 mL of solvent mixture (containing 200 mg of the dried algal sample) was sonicated for 10 minutes. Sonicated samples were centrifuged and solvents with extracted lipids were then collected into a pre-weighed dry vial. Such an extraction was repeated three times. The solvents from the vial were removed using an evaporator. Vials with dry lipids were weighed again to check the mass of the lipids.

### Fatty acid composition analysis

To validate the fatty acid composition, Gas Liquid Chromatography (GLC) was used according to the protocol listed as follows:

- Preparation of FAMEs: Fatty acid methyl esters (FAMES) were prepared by 2% H_2_SO_4_ methanol method. 2% H_2_SO_4_ in methanol was prepared by mixing 2 mL of H_2_SO_4_ (Certified ACS plus, Fisher Scientific) with 100 mL methanol (Chroma Solv, Sigma)^36^. A known amount of extracted total lipids was spiked with 20 μL of 1 mg/mL heptadecanoic acid (17:0, used as internal standard). The mixture was evaporated under nitrogen gas and to this 1 mL of 2% H_2_SO_4_ was added. The mixture was incubated at 86 °C for 1 h, cooled on ice for 5 min and subsequently neutralized by 0.5 mL 0.5% sodium chloride solution. FAMEs were extracted by the addition of 2 × 2 mL aliquots of hexane and then vortexed. The two layers were allowed to separate and the upper hexane layer was collected, and subjected to gas chromatographic analysis for identification and quantification of fatty acids.
- Gas chromatographic analysis of FAMEs: Analysis of FAMEs was performed on the Agilent 6890 N gas chromatography instrument coupled with an Agilent MS-5975 inert XL mass selective detector (Agilent technologies) in Electron Impact (EI) mode. Separation of fatty acids was achieved by injecting 2 μL of the FAMEs onto an 88% - Cyanopropyl aryl-polysiloxane column, HP88 (Agilent J & W Scientific, 30 × 0.25 mm × 0.25 μm). Splitless injection was performed with a constant carrier gas (helium) flow of 1 mL/min. Inlet temperature and transfer line temperatures were set at 200 °C and 180 °C respectively. Temperature programming was as follows: initial isotherm of 80 °C held for 1 min, raised to 90 °C at 1 °C/min, 90–250 °C at a rate of 6.1 °C/min, and held at the final temperature for 15 min. The MS ion source temperature was 230 °C and the Quadrupole temperature was 150 °C. Peak identification of fatty acids in the analyzed samples was carried out by comparison of chromatogram with mass spectral library (NIST) and against the retention times and mass spectra of Supelco 37 component FAME mix (Sigma-Aldrich, St Louis, MO, USA). The final result of different fatty acid components was calculated and expressed on a molar percentage basis according to the official methods of the Association of Analytical Chemists and the American Oil Chemists’ Society.

### Statistical analysis

Experimental values were determined in three independent experiments. Each experiment has been conducted in duplicates. All values regarding measurement and percentage of lipid content were expressed as mean and standard deviation (SD). The one-way analysis of variance (ANOVA) and Tukey multiple comparison post-tests were used. Differences less than 0.05 (p < 0.05) were considered statistically significant.

## Results

### Pulsed Wave for Enhancing Algae Lipid Production

**Figure 3:**
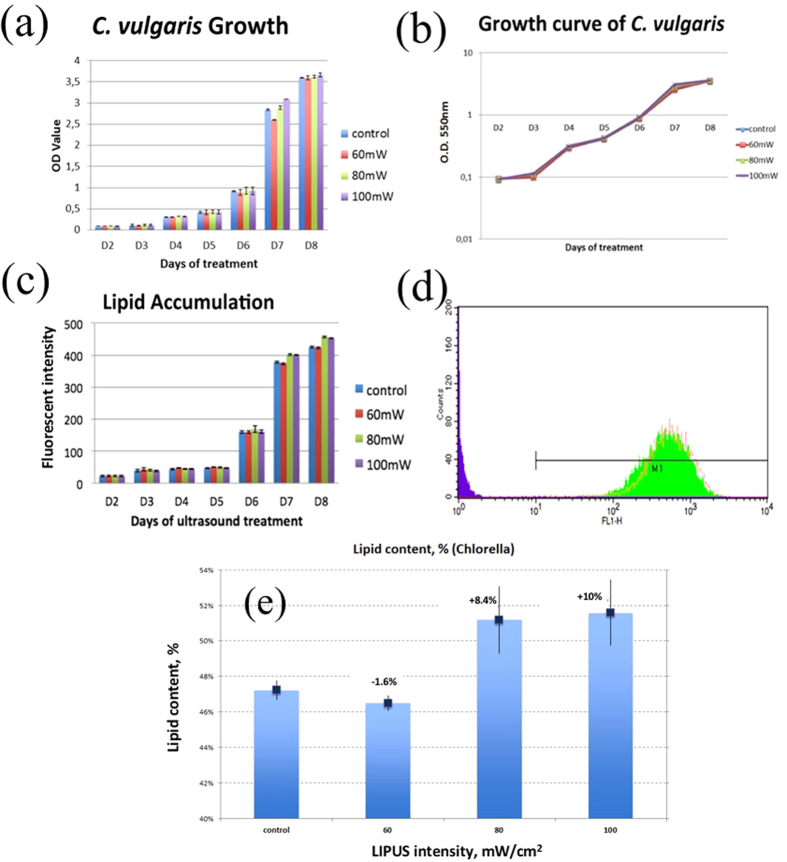
(**a**,**b)** Growth curve for *Chlorella vulgaris* in the LIPUS stimulation experiment based on OD_550_ measurements. Since cellular growth is exponential, growth curves are presented as a semi-logarithmic plot. (**c**) Lipid accumulation curve for *Chlorella vulgaris* based on fluorescence intensity measurements using flow cytometry. Note that the mean and standard deviation were obtained in 6 replicates (n = 6) (We have performed 3 independent experiments. Each experiment has been conducted in duplicates). A significant difference (p < 0.05) was observed on Day 7 and Day 8, when comparing samples with and without ultrasound treatments. **(d)** Results for lipid content in *C. vulgaris* cells with and without LIPUS stimulation following an 8-day cultivation cycle obtained using a flow cytometry histogram, where the green peak is for the control and red peak is for stimulated cells. [Control: no ultrasound stimulation]. Peak shift was observed on the FACS histogram for the stimulated sample. **(e)** Results for total lipid content per biomass in *Chlorella* cells with and without pulsed-wave stimulation in an 8-day cultivation cycle obtained using the extraction method.

To demonstrate the effect of LIPUS, *C. vulgaris* was chosen and mixotrophic growth conditions were used because it is the most commonly adopted growth condition in industry^13^. More specifically, phototrophic conditions were employed during the first four days of algae growth, and glucose as an organic substrate was then added on day five. Spectrophotometry with the OD_550_ as well as staining plus flow cytometry were used for measuring changes (cell growth and lipid accumulation) during the course of each experiment. LIPUS stimulation at the intensity levels of 80 mW/cm^2^ and 100 mW/cm^2^, for 15 minute per treatment every three hours has proven to be more efficient than the control (without ultrasound) in increasing lipid production of algal cells (refer to Fig. 3(a)). The experiment was repeated three times and each experiment has been conducted in duplicates to verify the reproducibility of results. Although no significant improvement in cell growth was observed, LIPUS stimulation yielded stable and repeatable increases in lipid production, up to 8.4% for 80 mW/cm^2^ and up to 10.0% for 100 mW/cm^2^ for *C. vulgaris* (refer to Fig. 3(b)) because all added energies were stored as lipid content rather than contributing to cell growth. However, samples stimulated with 60 mW/cm^2^ intensity did not show significant lipid increase compared to the control. The results indicate that the intensity greater than 80 mW/cm^2^ is required.

To track daily lipid accumulation, flow cytometry measurements coupled with fluorescent dyes were also performed for stained cells every day and then the results were compared with those obtained using the *Gravimetric* extraction method. Two dyes, Nile Red and BODIPY, were used for lipid staining. Nile Red staining required more effort due to difficulties with dye penetration into the cells, especially for *C. vulgaris*, which has thick cell walls. For flow cytometry measurements, because we took only 1 mL sample cell viability was not a concern, when it came to proper staining. To improve staining with Nile Red we used glycerol as a carrier when working with *C. vulgaris*. In addition to the carrier, sonication with heating was used to achieve better staining. On the other hand, it was generally much easier to stain with the BODIPY dye. When used with algal cells, the green fluorescent lipid probe BODIPY allowed for much easier staining. In addition, BODIPY required less time for sonication and milder heating conditions for *C. vulgaris* sample preparation. For the thin cell walls of *T. chuii* the BODIPY dye easily yielded perfect staining without any additional procedures by simply mixing a sample with the dye followed by 10 minutes of incubation in the dark. More importantly, the BODIPY dye gave much more stable and reliable results on the last day of the cycle that were similar to the ones obtained by the gravimetric extraction method. Based on these observations, BODIPY dye was used in real-time screening procedures to monitor the impact of ultrasound on lipid growth in algal cells. Fluorescence measurements using BODIPY showed the same increase (Fig. 3(c)), proving that results of lipid content measurements were in line with results of the *Gravimetric* method. Figure 3(e) shows total lipid content in *Chlorella* cells based on the gravimetric extraction method on the final day of cultivation cycle.

**Figure 4:**
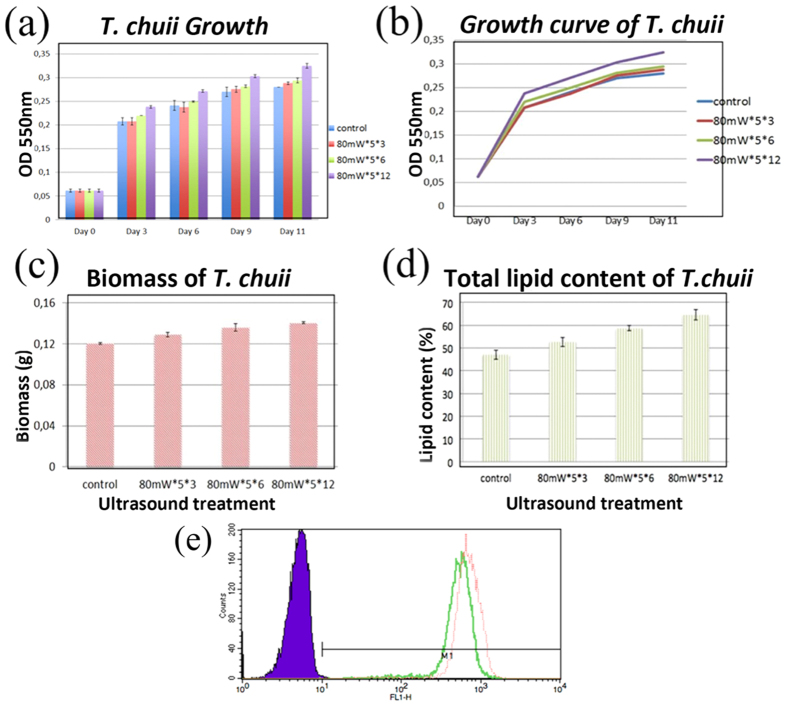
Effect of different LIPUS treatments on *T. chuii*. **(a,b)** Growth curve of *T. chuii* in F/2 medium. *T. chuii* was grown under the conditions described in the materials and methods section. Since cellular growth is exponential, growth curves are presented as semi-logarithmic plots. **(c) (d)** Total cell biomass and lipid content per dry biomass (%) are shown for different ultrasound conditions. Note that the mean and standard deviation were obtained in 6 replicates (n = 6) (We have performed 3 independent experiments. Each experiment was conducted in duplicates). Significant differences (p < 0.05) were observed both for biomass and total lipid when comparing samples with and without ultrasound treatments. **(e)** Results for lipid content in *T. chuii* cells with and without LIPUS stimulation following an 11-day cultivation cycle obtained using a flow cytometry histogram, where the green peak is for the control and red peak is for the ultrasound stimulated sample. The results show peak shift for stimulated sample. [Control: no ultrasound stimulation].

In addition to *C. vulgaris*, we have also tried to treat the seawater algae *T. chuii* using LIPUS. To measure the growth of *T. chuii*, 3 mL of culture was extracted every three days, and the OD at 550 nm was determined. The optimal set of ultrasound parameters was found to be 80 mW/cm^2^, 5 minutes and 12 times a day, which improved growth by 14% based on the OD_550_ measurement on day 12 (refer to Fig. 4(a,b)). Because *T. chuii* has thinner cell walls than *C. vulgaris,* the ultrasound stimulations given were milder, or 80 mW/cm^2^, 5 minutes 12 times a day instead of 100 mW/cm^2^, 15 minutes 8 times a day.

At the end of cultivation, the culture was harvested by centrifuging at 5000 rpm × g then freeze-dried for 24 hours. The dried samples were weighed to measure their biomass. Total lipid was extracted using the chloroform–methanol (2:1 ratio) extraction method described^37^. Conditions of 80 mW/cm^2^, 5 minutes and 12 times a day were found to induce the highest growth and higher lipid content. The improvement in growth measured from dry biomass is 17%, which is also very close to the value calculated from the OD at 550 nm. The increase of total lipid content after normalized by biomass is about 24% with this condition (refer to Fig. 4(c,d)). We also used flow cytometry measurements to validate the results (refer to Fig. 4(e)).

## Discussions

### Cost Analysis

Instead of reporting a basic scientific discovery, this article focuses on a new technological development. From the technological viewpoint, we have successfully translated the proof-of-concept design (in shake flasks) to bench scale (in bioreactors). The one-time installation cost (including a LIPUS generator and an ultrasound transducer) for a 1 L spinner-flask bioreactor is less than $50 US Dollars. The routine cost to trade10 ~ 20% increase in lipid content is the cost of electricity. According to our previous discussion, the following are the treatment conditions:

- *T. chuii*: 80 mW/cm^2^, 5 minutes, 12 times a day, and
- *C. vulgaris*: 100 mW/cm^2^, 15 minutes, 8 times a day.

The power efficiency of our LIPUS driving circuit is about 40%, and the surface area of a transducer is 3.5 cm^2^. Therefore, the total energy consumption of the LIPUS device to stimulate *T. chuii* is ((80 mW/cm^2^*3.5 cm^2^)/40%) *5 minutes*12 times/per day*8 days = 700 mJ/s*3600 s/per day*8 days = 0.7 J* 28800 = 20160 J. Since 1kilowatt-hour (kWh) = 3600000 J, then 20160 J = 0.0056 kWh. Similarly, the total energy needed to stimulate *C. vulgaris* using LIPUS is ((100 mW/cm^2^*3.5 cm^2^)/40%) *15 minutes *8 times/per day*8 days = 14 Wh = 0.014 kWh. According to the report of electricity rates in North America (http://www.gov.mb.ca/jec/invest/busfacts/utilities/compare.html), the average cost of electricity is about $0.10 US Dollar per kWh. Or, the total cost needed for *T. chuii* is 0.0056 kWh*$0.1/kWh = $0.00056, and $0.0014 for *C. vulgaris*, respectively. The benefit gain is obvious.

### Shear Stress Induced by Pulsed Wave can Increase Lipid Accumulation

**Figure 5:**
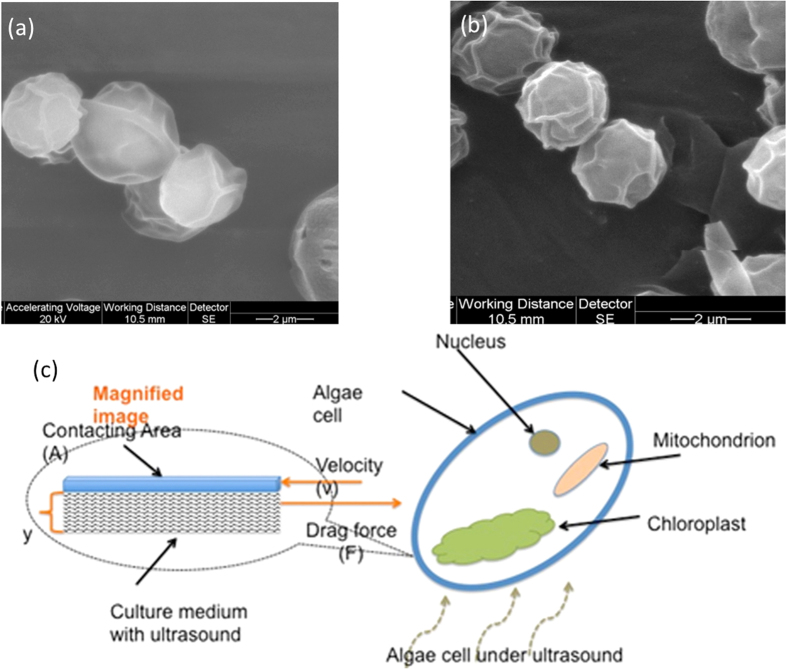
(**a**,**b**) SEM images of *Chlorella vulgaris* cells without (**a**) and with (**b**) pulsed wave, respectively. **(c)** Schematic of proposed mechanism for ultrasound-enhancement of algae lipid production. Appropriate amounts of shear stress induced by LIPUS can promote algal lipid accumulation.

We chose pulsed ultrasound because we have observed that the pulsed wave can induce physiological changes (higher growth rates and protein expression)^38^,^39^. Although *Chlorella vulgaris* has a thick cell membrane, if we look closely, we can still observe that the algae cell membrane becomes more wrinkled into irregular folds Fig. 5(b) after the ultrasound treatments compared to the one without the treatments (Fig. 5(a)). In other words, the ultrasound effectively increases its specific surface area for respiration and cell uptake (the cell surface recovers after pulsed-wave is removed). Based on our previous studies, 40 mW/cm^2^ to 60 mW/cm^2^ stimulation intensity is required for mammalian cells^38^,^39^. However, the degree of this effect depends on the thickness of the cell wall. For thick cell walls more ultrasound treatment is required to achieve the same effect^40^. In this study, we selected 60 mW/cm^2^ to 100 mW/cm^2^ for algae cells.

Ultrasound stimulation induces shear stress on algae cells, which results in more algal lipid. Considering the culture medium as a non-compressible Newtonian fluid, the shear stress (*F/A*) is proportional to the velocity gradient (*dv/dy*) (shown in Fig. 5(c)). Or, 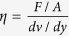, where *η* is the viscosity of the culture medium. The cell of area *A* is moved with the constant velocity *v* on a layer of the culture medium with the thickness of *y.* The force (*F*) is the drag exerted by the liquid under LIPUS stimulation (refer to Fig. 5(c)). The shear stress varies depending on the viscosity, the thickness of cell wall and the contact area between cells and culture medium. The shear stress induced by ultrasound can also cause microstreaming^41^, which induce air microbubbles around living cells due to an abundance of dissolved atmospheric gasses in the cell culture media. These microbubbles are encapsulated between the bilayer of cell membrane can increase cell permeability when they burst. Based on our previous studies^38^, Lactate Dehydrogenase (LDH) plays an important role in cellular respiration, and it was used in the test cell permeability changes. An increase in LDH uptake was observed in pulsed-wave treated cells vs. the control. Suitable amounts of shear stress induced by LIPUS, which can vary for different cell type, can increase protein expression^38^,^40^. The same effect affects algal cells, or pulsed wave can increase cell permeability and help cell metabolism, and thus biomass/lipid content accumulation can be achieved. *Chlorella vulgaris* have thicker cell walls than *T. chuii*, hence reduced shear stress explains why the LIPUS stimulation is less efficient in producing lipid compared to *T. chuii*. However, increasing shear stress too much can lead to cell death. Therefore, screening for the appropriate ultrasound simulation condition is crucial. The difference between the ultrasound stimulated and the control samples was also observed in cell count: after only 3 days of stimulation the amount of cells in the control sample was 1.85*10^6^, while the stimulated samples had almost doubled in the number of cells: 3.04*10^6^ for 60 mW/cm^2^, 4.02*10^6^ for 80 mW/cm^2^ and 4.39*10^6^ for 100 mW/cm^2^. After eight days of LIPUS stimulation, the cells, especially in treated samples ran out of nutrients and started to consume their own lipids. As a result, eight-day growth cycle was chosen for all experiments.

### Quality of Lipids Remains Unchanged After Pulsed Wave Stimulation

**Figure 6:**
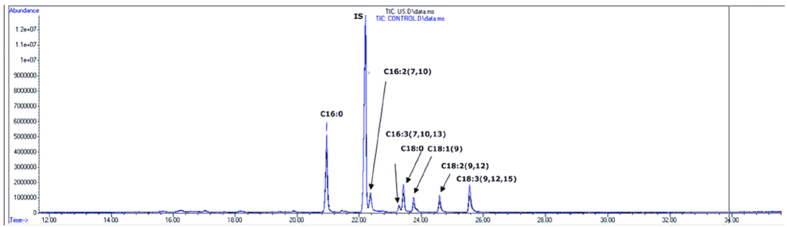
Gas Liquid Chromatography (GLC) analysis results for *Chlorella* to verify that there were no changes in lipid composition induced by pulsed wave stimulation. The peak identification and assignment are labeled in the figure. The blue line represents the control sample without pulsed wave stimulation while the black line denotes the sample which has undergone pulsed wave stimulations. Both lines are overlapped with each other, which means that pulsed wave stimulations do not alter the fatty acid composition.

Experiments to verify that fatty acid composition remains unchanged after pulsed wave stimulation were performed using lipids extracted from *Chlorella*. The methods used for comparison were GLC. The results show that the fatty acid composition remains the same with or without pulsed wave stimulations (refer to Table 1 and Fig. 6).

### Accurate and Fast Daily Screening of Algae Lipid Content

Lipid accumulation by algae cells is not a rapid process and the size of the lipids in algae cells changes gradually increasing within the 1 μm range during growth cycle. This is why a method for real-time screening lipid increase needs to be accurate and sensitive (detecting even tiny fluctuations in lipid concentrations). The traditional *Gravimetric* method cannot be chosen because it takes more than 24 hours of sample preparation and a large sample volume for accuracy. Consequently, an accurate and fast daily screening method is needed.

**Figure 7:**
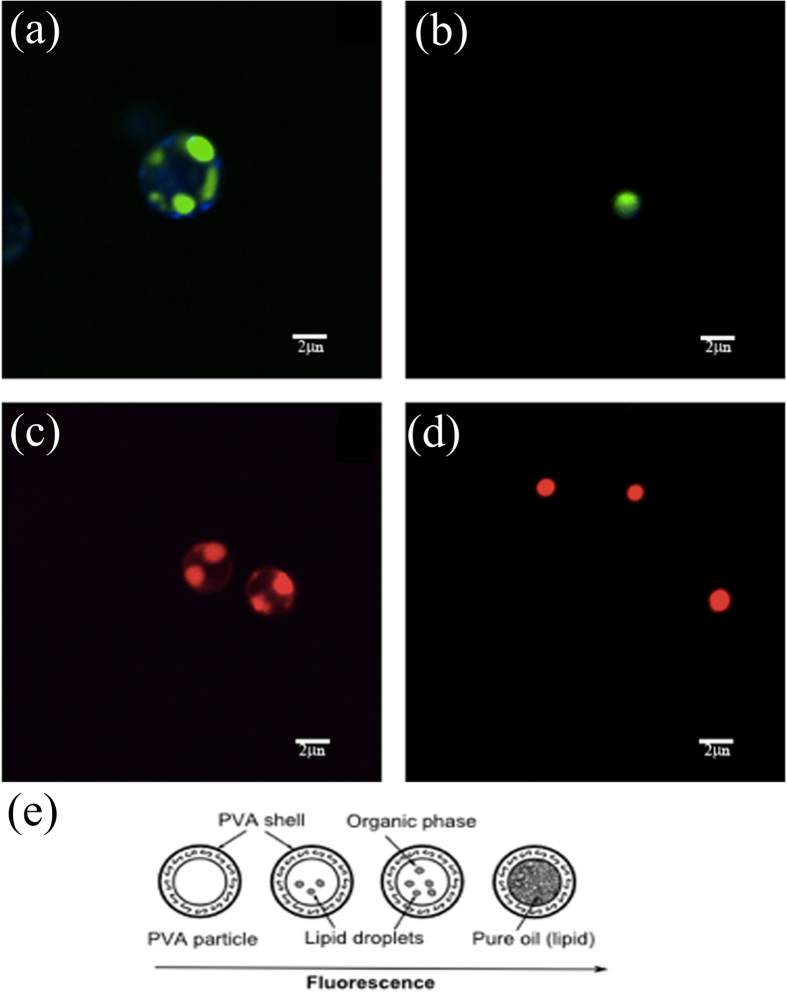
Confocal laser scanning microscopy images: (**a,c**) *C. vulgaris* cells with lipids stained by (**a**) BODIPY staining, (**c**) Nile red staining. (**b,d**) Oil particles from o/w emulsion alone with lipids stained by (**b**) BODIPY staining, (**d**) Nile Red staining. (**e**) Schematic diagram of the lipid droplets in emulsion.

Daily optical density (OD) measurements can be used to monitor biomass increase, but a technique that can monitor lipid accumulation in algae cells on a daily basis has yet to be developed. Such an evaluation is very useful in finding the optimal and most cost-efficient culture condition by using a very small amount of culture sample (<1 mL). Although lipid content measurements based on the conventional *Gravimetric* extraction to determine lipid content have been widely used^42^, such a method requires 24 hours of sample preparation, and therefore unsuitable for real-time monitoring. Lipid staining with fluorescent dyes has previously been reported^43^ as a method widely used to screen microalgae species in both fresh water and brackish water for their potential to accumulate lipids. For instance, fluorescent lipid probes, like Nile Red, coupled with the flow cytometry technique have been successfully used to stain cell lipids in order to qualitatively select the most lipid productive cells^43^,^44^. Because these cells were used for culturing after selection, cell viability was important after staining. Studies to improve staining efficiency using Nile Red dye without damaging cells have been published recently^45^,^46^. The research shows that the use of carriers can achieve better dye penetration into algae cells while keeping them alive. Cell viability, however, is not important for lipid accumulation measurements. Accuracy, repeatability and easy staining protocol as well as small sample culture volume are more important criteria. Although a new dye BODIPY has recently been suggested as an alternative lipid staining method for algal cells^45^, its full potential for lipid quantification has not been fully explored. In this article, we suggest a new lipid staining method. Both BODIPY and Nile Red dyes were used for lipid staining in order to select an appropriate dye that meets the needs of real-time lipid content measurements while yielding more reliable results. We have used lipid droplets in emulsion as the model system to evaluate the two dyes. While the size of lipid droplets varies much in the cells, in the emulsion we can create a system with lipid particles of known size and see how fluorescence reflects the increase of lipid content. Particles were made using linolenic acid, one of the natural lipids accumulated in algae (e.g. *C. vulgaris*) cells^47^. To allow for easy staining and also avoid interference from confounding factors, such as how different staining protocols can affect the accuracy of results, only a thin PVA shell was used to cover lipid particles. Figure 7(e) shows a schematic diagram of the oil particle structure used as a model system for dye selection, where the oil content in the core of the particles (inside the PVA shell) increases from left to right. The staining procedure was similar to that for algae staining except that (i) particles did not require any carrier for the dye; and (ii) neither sonication nor heating was needed for the dye to penetrate the PVA shell and to stain the lipids. The procedure was fast and simple. Particles of different sizes within a range of 2.85 μm to 3.85 μm were obtained using different speeds of the homogenizer to form an emulsion (the higher the speed, the smaller the size of the droplets in the emulsion). Emulsions prepared in such a way yielded a narrow size distribution of lipid particles as measured using DLS. The data show that the size difference of the lipid droplets in different emulsion samples (obtained by different emulsification speeds) was within a range of 1 μm. Such a size difference can naturally occur in algae cells, when lipid content increases during the growth cycle. The size variation also provides an opportunity to evaluate sensitivity of our screening method.

**Figure 8:**
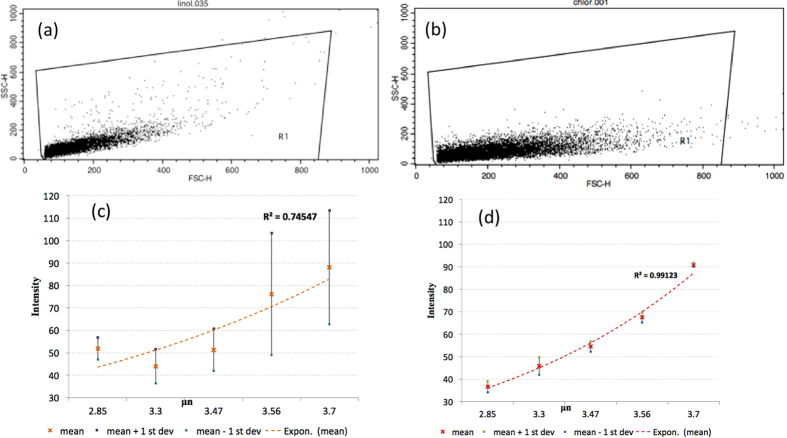
Flow cytometry measurements of stained algae cells **(a)** and stained oil particles **(b)**, showing similar FACS histograms for both cases. 10,000 particles were used to reduce statistical deviation. Fluorescence intensity obtained for different sizes of oil particles stained with BODIPY dye (**c**) and Nile Red Dye (**d**) showing better accuracy with BODIPY staining.

Similar to how flow cytometry is used to measure fluorescence per algal cell, flow cytometry can also be used to mesasure fluorescence intensity per lipid particle after emulsification. The experimental results showed that larger sized particles correlated to higher lipid content, indicated by higher fluorescence intensity after staining. These results were consistent with our expectation. The size of oil droplets in our emulsions was close to the size of lipid droplets in the algae cells. This can be seen on the confocal images of both stained particles and stained cells using either BODIPY or Nile Red dyes (shown in Fig. 7(a–d)). The size of oil particles was similar with the size of the lipids in the algae *C. vulgaris* cells at the end of their growth cycle. 10,000 particles were used in flow cytometry measurement to reduce statistical deviation. The flow cytometry machine counts the number of particles and measures their fluorescence, analogous to measurements performed on cells. Flow cytometry histogram plots of stained algae cell and stained oil particles are shown in Fig. 8(a,b), respectively. Along the Y-axis (HSSC-H) is the side-scattered light, which is proportional to the internal complexity. Along the X-axis (FSC) is forward scattered light, which is proportional to the size or the surface of cells or particles. These two histogram plots look very similar except that particle size distribution is narrower than that of algae cells. Therefore, our model system with emulsions could be used for selecting the more accurate dye for lipid content measurement (BODIPY vs. Nile Red). When the emulsions were used for calibration, repeatable and stable results were obtained by using BODIPY dye, while the Nile Red dye produced deviations too great to be used (refer to Fig. 8(c,d)).

The lipid content increase measured by the gravimetric extraction matched the results using BODIPY staining measured by flow cytometry on the final day of cultivation cycle. Therefore, BODIPY staining coupled with flow cytometry allowed quantitative measurement of the difference between samples. However, fluorescence measurements with Nile Red had poor stability and could not be used quantitatively because some measurements showed lipid content increase up to 75% or more between samples, which could not be true according to our extraction measurements. Although the Nile Red dye was extensively used for lipid staining^35^,^47^, in our case better repeatability and more accurate results were obtained using the BODIPY dye.

## Conclusions

With the selection of the best algal strain for lipid accumulation or improvement of media, the pulsed ultrasound stimulation is a complementary physical method to induce physiological changes of algae cells. In this study, a pulsed wave stimulation technology was designed to increase efficiency and reduce cost of lipid production by microalgae. Both freshwater algae *C. vulgaris* and seawater algae *T. chuii* were studied. Pulsed wave stimulation was shown to yield an increase in lipid content up to 20%, which makes the technology very attractive. A contribution of this paper is proposing emulsion preparation techniques for creating a set of flow cytometry standards for lipid measurement. The tests of two different fluorophores on polymer particles showed that BODIPY could provide better accuracy of measurements compared to Nile Red dye. Because this fluorescent staining method for monitoring daily lipid accumulation requires only small volumes of culture sample, it is ideal for research labs or manufacturers to assess the increase of algal lipids and to optimize culturing conditions. We are developing a pulsed wave stimulation embedded in large-scale systems, such as open ponds, raceways, and photo-bioreactors, for microalgae biomass production.

## Additional Information

**How to cite this article**: Savchenko, O. *et al*. Algal Cell Response to Pulsed Waved Stimulation and Its Application to Increase Algal Lipid Production. *Sci. Rep.* **7**, 42003; doi: 10.1038/srep42003 (2017).

**Publisher's note:** Springer Nature remains neutral with regard to jurisdictional claims in published maps and institutional affiliations.

## Figures and Tables

**Table 1:** Lipid composition analysis for samples with and without pulsed wave stimulation.

		Fatty acid composition of total lipid fractions for the Control					
	(mol % of total fatty acids)						
16:0	16:2 (7,10)	16:3 (7,10,13)	18:0	18:1 (9)	18:2 (9,12)	18:3 (9,12,15)
34.8 ± 6.9	10.8 ± 0.1	11.0 ± 4.0	3.4 ± 0.1	6.1 ± 2.3	15.1 ± 8.6	18.4 ± 3.4
	**Fatty acid composition of total lipid fractions for the Treated Sample**					
	**(mol % of total fatty acids)**					
16:0	16:2 (7,10)	16:3 (7,10,13)	18:0	18:1 (9)	18:2 (9,12)	18:3 (9,12,15)
37.3 ± 5.7	10.7 ± 2.2	8.4 ± 6.2	3.2 ± 1.2	8.5 ± 0.7	16.1 ± 11.4	15.4 ± 2.3
